# Supplementary material for: Traumatic dental injuries in permanent teeth among Arab children: prevalence, and associated risk factors—a systematic review and meta-analysis
Source: PeerJ. 2024 Dec 24;12:e18366. doi: 10.7717/peerj.18366 (PMC11674148; doi:10.7717/peerj.18366)
Supplement: Supplemental Information 1 [file peerj-12-18366-s001.doc]

**RESEARCH RATIONALE**

A systematic review and meta-analysis of dental trauma in the Arab population may reveal the following unfilled areas of study:

**Research is limited**: It is probable that there is a lack of original studies on oral trauma in the Arab population in the published literature. Lack of knowledge, limited funding, or cultural factors affecting research priorities might all be to blame.

**Heterogeneity of methodology**: It may be difficult to compare and combine the results of the existing research because they may have used different methodologies, diagnostic standards, and sampling procedures. The validity and dependability of the meta-analysis may be impacted by this heterogeneity.

**Geographic representation:** The occurrence of dental trauma may range between various Arab nations or regions. The generalizability of the results to the entire Arab population may be constrained by the studies' insufficient representation of certain Arab countries or areas.

**Language bias:** Studies published only in certain languages, such as English, may be included, leaving out pertinent studies published in other languages. The review's thoroughness and representativeness may suffer as a result of this prejudice.

**Disparities by gender and age:** Dental trauma may occur at a different rate across different age groups and sexes. If the existing research does not adequately represent the diverse age ranges and gender distribution within the Arab population, prevalence figures may be erroneous and distorted.

**Lack of standardized reporting:** In the known research, there are no standardized reporting processes or guidelines, which could result in inaccurate or inconsistent data reporting. As a result, it may be difficult to accurately collect and synthesize data throughout the systematic review process.

A thorough and methodical strategy is needed to fill in these research gaps. To find a wider variety of research, it entails undertaking a thorough literature search across numerous databases, including both English and non-English sources. Additionally, efforts can be made to persuade researchers to adopt the standardized methodology and reporting practices and to perform studies that especially target oral trauma in the Arab community.

The prevalence of oral trauma in the Arab population can be better understood by solving these research gaps, which can also help to inform clinical practice, guide policy decisions, and identify areas that require further research.
